# Bringing Loyalty to E-health: Theory Validation Using Three Internet-Delivered Interventions

**DOI:** 10.2196/jmir.1837

**Published:** 2011-09-24

**Authors:** Rik Crutzen, Dianne Cyr, Nanne K de Vries

**Affiliations:** ^1^Maastricht University/CAPHRIMaastrichtNetherlands; ^2^Simon Fraser UniversitySurrey, BCCanada

**Keywords:** e-Loyalty, adherence, attrition, user perceptions, theory, Internet, interventions

## Abstract

**Background:**

Internet-delivered interventions can effectively change health risk behaviors, but the actual use of these interventions by the target group once they access the website is often very low (high attrition, low adherence). Therefore, it is relevant and necessary to focus on factors related to use of an intervention once people arrive at the intervention website. We focused on user perceptions resulting in e-loyalty (ie, intention to visit an intervention again and to recommend it to others). A background theory for e-loyalty, however, is still lacking for Internet-delivered interventions.

**Objective:**

The objective of our study was to propose and validate a conceptual model regarding user perceptions and e-loyalty within the field of eHealth.

**Methods:**

We presented at random 3 primary prevention interventions aimed at the general public and, subsequently, participants completed validated measures regarding user perceptions and e-loyalty. Time on each intervention website was assessed by means of server registrations.

**Results:**

Of the 592 people who were invited to participate, 397 initiated the study (response rate: 67%) and 351 (48% female, mean age 43 years, varying in educational level) finished the study (retention rate: 88%). Internal consistency of all measures was high (Cronbach alpha > .87). The findings demonstrate that the user perceptions regarding effectiveness (beta_range_ .21–.41) and enjoyment (beta_range_ .14–.24) both had a positive effect on e-loyalty, which was mediated by active trust (beta_range_ .27–.60). User perceptions and e-loyalty had low correlations with time on the website (*r*_range_ .04–.18).

**Conclusions:**

The consistent pattern of findings speaks in favor of their robustness and contributes to theory validation regarding e-loyalty. The importance of a theory-driven solution to a practice-based problem (ie, low actual use) needs to be stressed in view of the importance of the Internet in terms of intervention development. Longitudinal studies are needed to investigate whether people will actually revisit intervention websites and whether this leads to changes in health risk behaviors.

## Introduction

Internet-delivered interventions can effectively change health risk behaviors (eg, lack of physical activity, low consumption of fruit, cigarette smoking, and excessive alcohol consumption) [1]. However, the actual use of these interventions by the target group once they access the website is very low [2,3]. For example, server statistics of an intervention promoting heart-healthy behaviors showed that 285,146 visitors from unique internet protocol (IP) addresses landed on the home page in a 36-month period, but 56.3% of these left the intervention website within 30 seconds [4]. This finding touches on the critical issue in Internet-delivered interventions: how can behavior ever be changed if people are not exposed or are only briefly exposed to the actual intervention? Therefore, it is relevant and necessary to focus on factors related to use of an intervention once people arrive at the intervention website. These factors relate to the *visitor* (eg, people’s motivation to be healthy [5,6]) as well as the *intervention* website (eg, visual complexity of the homepage). Two recently published systematic reviews provide a detailed overview of factors used by current *interventions* to stimulate use of intervention websites [7,8]. Our study focused on perceptions of *visitors* resulting in a user experience [9,10].

User experience refers to what a person thinks and feels during and after exposure to a website [11]. The main idea is that a positive user experience leads to increased website use. User experience consists of cognitive and affective perceptions [12]. Cognitive perceptions are rational in nature and are induced by utilitarian or cognitive motives. Affective perceptions are emotional in nature and are induced by hedonic or affective motives [13]. Previous studies demonstrated the importance of these perceptions regarding intention to use a technology [14] and to visit a website again [12]. We designed our study on the basis of these findings and applied them to loyalty regarding intervention websites in the field of eHealth (ie, e-loyalty). Besides visiting an Internet-delivered intervention again, e-loyalty also consists of recommending an Internet-delivered intervention to others. The latter is based on previous research indicating that word-of-mouth is an effective strategy to improve use of Internet-delivered interventions [15,16]. A background theory for e-loyalty is still lacking for Internet-delivered interventions. Although previous studies did explicitly describe the theory used to develop the content of Internet-delivered interventions, these theories primarily related to behavior determinants or behavior change [8,17]. Theory development regarding e-loyalty is highly needed to increase the public health impact of Internet-delivered interventions. Therefore, in this study we propose and validate a conceptual model.

**Figure 1:**
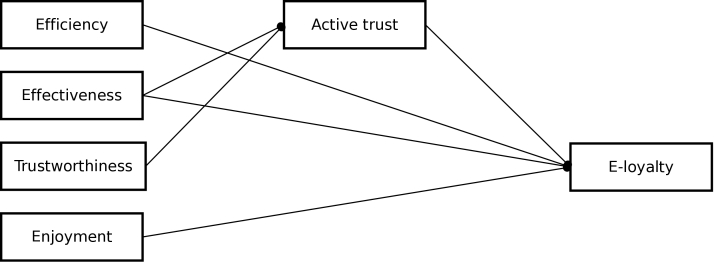
Conceptual model.

To systematically constitute the proposed conceptual model, we describe conceptual definitions and their relationship with e-loyalty [18]. Terminology that is used within the conceptual model (ie, key user perceptions) is derived from other fields such as e-commerce. Although these terms can have a different meaning within public health, we chose to use the same terminology as in previous studies in other fields to avoid further confusion. The key user perceptions in the conceptual model are efficiency, effectiveness, trustworthiness, enjoyment, and active trust [12]. *Efficiency* refers to easy search of and access to the information provided, and *effectiveness* refers to the quality of that information (eg, in terms of relevance) [19]. These cognitive perceptions have parallels with perceived ease of use and perceived usefulness in the technology acceptance model, but are applicable in a broader context [20]. The positive effect of these cognitive perceptions on e-loyalty has been demonstrated in, for example, e-service environments [21]. The idea that a positive user experience leads to e-loyalty applies not only to cognitive perceptions, but also to affective perceptions [5,22]. These affective perceptions are often referred to as *enjoyment* [23] and have been demonstrated to have a positive effect on e-loyalty in, for example, e-commerce [24]. *Trustworthiness* is defined as the believability of the provided information and refers to both cognitive and affective perceptions: it is based on a cognitive process (eg, rational reasons) and an emotional base (eg, a strong positive affect for the trustee) [25]. It has been demonstrated to have a positive effect on e-loyalty in, for example, online shopping [26,27]. *Active trust* might be a working mechanism leading to e-loyalty [28]. Whereas trustworthiness refers to the believability (eg, “I trust the information presented on this website”), active trust refers to the confidence in acting on the provided information (eg, “I would act on the information presented on this website”). Active trust has been proven to be the primary intermediate associated with e-loyalty [26,29]. In line with the study of Cugelman and colleagues [28], we expected active trust to mediate the impact of trustworthiness and effectiveness on e-loyalty. This resulted in the following hypotheses to be tested in a new context lacking a background theory: the field of eHealth (Figure 1).

### Hypothesis 1

H1a Efficiency has a positive effect on e-loyalty.

H1b Effectiveness has a positive effect on e-loyalty.

H1c Enjoyment has a positive effect on e-loyalty.

### Hypothesis 2

H2a Active trust mediates the relationship between effectiveness and e-loyalty.

H2b Active trust mediates the relationship between trustworthiness and e-loyalty.

## Methods

To improve the external and ecological validity, we included 3 generally available, Internet-delivered interventions. The interventions were certified according to the guidelines of the Dutch recognition system for health promotion interventions [30]. The quality assessment of health promotion interventions is supervised by the Netherlands Institute for Public Health and the Environment (interventions aimed at adults) and the Netherlands Youth Institute (interventions aimed at youth) [31]. We included interventions from all levels of recognition (theoretically sound, probable effectiveness, and established effectiveness; inspired by the UK Medical Research Council’s evaluation framework for complex interventions) in this study. The first intervention, registered by the Consumer and Safety Foundation (Netherlands), was theoretically sound and is concerned with prevention of sports injuries (intervention 1 [32]). The second intervention, registered by the Netherlands Institute of Mental Health and Addiction, was probably effective and is concerned with drinking less alcohol (intervention 2 [33]). The third intervention, registered by the Netherlands Institute of Mental Health and Addiction, was effective and is intended for people feeling gloomy or having mild depressive complaints (intervention 3 [34]). We must stress that these were all primary prevention interventions aimed at the general public. In other words, these interventions were not targeted at diagnosing (secondary prevention) or treating (tertiary prevention) health problems related to health risk behaviors, but at people who did not yet have these problems. Hence, these interventions were deemed of interest to the general public.

### Participants

Participants were recruited through a research panel of a Dutch Internet research agency [35]. From this panel, we invited through email a stratified sample of 592 potential participants to take part in this study. This sample was representative of the Dutch population above 18 years, taking into account gender, age, and level of education. Of those invited, 397 clicked on the link in the invitation email to start the study (response rate: 67%) and 351 finished the study (retention rate: 88%). There was no selective dropout regarding gender (n = 592, χ^2^_1_ = 3.2, *P* = .08), but those who dropped out were somewhat younger (40 vs 43 years, *t*_590_ = 2.86, *P* = .004) and differed in terms of level of education (n = 592, χ^2^_1_ = 10.9, *P* = .004). The final sample consisted of 48% (169/351) women; the average age was 43 (SD 13) years. In terms of level of education, 30% (107/351) of the participants had a low level of highest completed education, 35% (122/351) an intermediate level, and 35% (122/351) a high level (according to the definitions of Statistics Netherlands).

### Procedure

The study consisted of 3 blocks (ie, 3 intervention websites and related measurements) that were presented at random to each participant. In each block participants were exposed to 1 of the 3 intervention websites described above, and subsequently participants completed the measures described in the measurements section. Participants were asked to assess several websites. It was stressed that there were no right or wrong answers and they could base their opinion on their first impression. The reason behind this was to prevent participants from thoroughly studying the intervention website, and to mimic a real-life situation in which the time being exposed to and willing to investigate an intervention website is often limited [4]. On average, participants took 17 minutes to complete the full study (eg, exploring the intervention websites and completing related measurements). Participants received credit points for participating in the study, for a value of €1.95. 

### Measurements

Directly after exposure to each intervention website, participants indicated whether they had seen the website before. For each intervention website, data from participants who indicated that they had seen website before were removed, because their perceptions and loyalty might have been based on the previous exposure to the intervention website. This concerned 8 (different) participants per intervention website and results did not differ if their data were included. Subsequently, participants completed the following validated measures after being exposed to each intervention website.

*E-loyalty:* intention to visit the website again (eg, “It is likely that I will visit the website again in the future”) and whether participants would recommend the website to others (“It is likely that I will recommend this website to others “) were assessed by 3 items each [36]. Items were answered on a 7-point Likert scale ranging from “strongly agree” to “strongly disagree.”

*User perceptions:* efficiency (eg, “I was able to access the information quickly on this website”), effectiveness (eg, “The website provided me with relevant information about...”), trustworthiness (eg, “I trust the information presented on this website”), enjoyment (eg, “I found my visit to this website enjoyable”), and active trust (eg, “I would act on the information presented on this website if needed”) were assessed by 3 items each [21,37]. Items were answered on a 7-point Likert scale ranging from “strongly agree” to “strongly disagree.”

Two native speakers (RC and an assistant) translated all items into Dutch and discussed semantic similarity until reaching a consensus. Besides these self-reported measures, time on each intervention website was assessed by means of server registrations.

### Analyses

First, using Predictive Analytics SoftWare Statistics (version 18.0; IBM Corporation, Somers, NY, USA), we conducted correlation and reliability analyses for each intervention website separately. Subsequently, using Mplus (version 5; Muthén & Muthén, Los Angeles, CA, USA), we constructed structural equation models to test the hypotheses per intervention website. First of all, we tested the hypothesized conceptual model: intention to visit again and recommending to others were regressed on efficiency, effectiveness, and enjoyment; active trust was regressed on effectiveness and trustworthiness. Subsequently, we added paths to the conceptual model based on modification indices, which are chi-square distributed, implying that a modification index larger than 3.84 indicates that adding the suggested path will significantly improve model fit. The reason to include paths beyond the hypotheses was to explore whether unanticipated relationships might explain variance in e-loyalty and, hence, contribute to theory development. The criterion for accepting or rejecting a hypothesis was a significant pattern across all 3 models. A level of significance of .05 was used for the relationships within the model.

Comparative fit index (CFI), Tucker-Lewis index (TLI), root mean square error of approximation (RMSEA), and standardized root mean square residual (SRMR) were used as fit indices for each model. CFI and TLI are goodness-of-fit indices, where larger values signal better fit. Values over .95 indicate close fit. RMSEA and SRMR are goodness-of-fit indices, where larger values signal worse fit. Rules of thumb for close fit are RMSEA ≤ .05 and SRMR ≤ .09 [38,39].

## Results

Tables 1–3 show the results of correlation and reliability analyses. Internal consistency of all measures was high (Cronbach alpha > .87). Overall, correlations between user perceptions and e-loyalty were high (*r*_range_ .44–.84). User perceptions and e-loyalty have low correlations with time on the website (*r*_range_ .04–.18).

**Table 1:** Correlation matrix intervention 1 (N = 343)

	Alpha	Mean	SD	1	2	3	4	5	6	7	8
1. Efficiency	.98	4.7	1.6	–	.68	.72	.71	.61	.58	.60	.04
2. Effectiveness	.95	4.4	1.6		–	.79	.77	.82	.75	.76	.13
3. Trustworthiness	.97	4.7	1.4			–	.73	.72	.64	.66	.16
4. Enjoyment	.99	4.1	1.7				–	.79	.74	.75	.09
5. Active trust	.94	4.2	1.7					–	.76	.76	.10
6. Intention to visit again	.89	3.6	1.7						–	.84	.09
7. Recommend to others	.95	4.0	1.7							–	.14
8. Time on website (minutes)	–	3:06									–

**Table 2:** Correlation matrix intervention 2 (N = 343)

	Alpha	Mean	SD	1	2	3	4	5	6	7	8
1. Efficiency	.97	4.8	1.5	–	.62	.63	.60	.57	.49	.58	.12
2. Effectiveness	.91	4.3	1.5		–	.71	.67	.76	.63	.70	.16
3. Trustworthiness	.96	4.6	1.4			–	.66	.68	.50	.61	.14
4. Enjoyment	.98	4.0	1.5				–	.71	.62	.67	.09
5. Active trust	.91	4.1	1.6					–	.71	.74	.16
6. Intention to visit again	.87	3.3	1.7						–	.77	.18
7. Recommend to others	.94	4.0	1.7							–	.16
8. Time on website (minutes)	–	1:28									–

**Table 3:** Correlation matrix intervention 3 (N = 343)

	Alpha	Mean	SD	1	2	3	4	5	6	7	8
1. Efficiency	.98	4.7	1.5	–	.63	.61	.57	.57	.44	.57	.09
2. Effectiveness	.95	3.9	1.7		–	.73	.72	.77	.72	.78	.14
3. Trustworthiness	.97	4.4	1.4			–	.70	.70	.56	.69	.07
4. Enjoyment	.98	4.0	1.6				–	.79	.70	.75	.11
5. Active trust	.94	3.8	1.7					–	.74	77	.09
6. Intention to visit again	.91	3.3	1.7						–	.82	.10
7. Recommend to others	.96	3.7	1.8							–	.14
8. Time on website (minutes)	–	3:10									–

**Figure 2:**
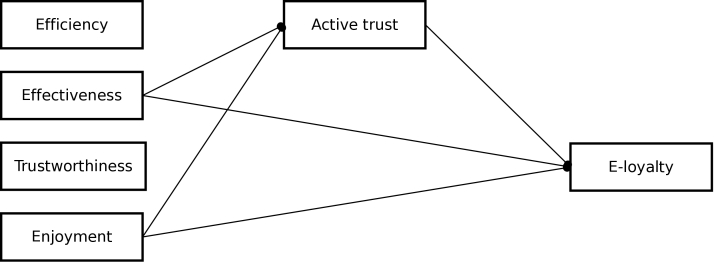
Extended Model.

Table 4 shows the results of the structural equation models when testing the conceptual model. H1a was rejected; efficiency did not have a positive effect on e-loyalty. H1b and H1c were confirmed; both effectiveness and enjoyment had a positive effect on e-loyalty. H2a was also confirmed; active trust mediated the relationship between effectiveness and e-loyalty. Results for H2b were mixed, because the relationship between trustworthiness and active trust differed in terms of being significant and standardized betas [40,41]. Therefore, the relationship between trustworthiness and active trust was included when adding paths to the conceptual model based on modification indices. The only path that was added to the conceptual model was the relationship between enjoyment and active trust. Modification indices (respective values of 50.27, 39.15, and 72.62) suggested the addition of this path to each model representing an intervention website. Table 5 shows the results of the structural equation models when testing this extended model. The results were similar to the conceptual model: H1a was rejected and H1b, H1c, and H2a were confirmed. H2b, however, was rejected; active trust did not mediate the relationship between trustworthiness and e-loyalty, because there was no relationship between trustworthiness and active trust. Unanticipatedly, but consistently, the positive effect of enjoyment was mediated by active trust. All fit indices indicated good fit for the extended model. Figure 2 shows the extended model resulting from the analyses for all 3 intervention websites.

**Table 4:** Results of conceptual model (figures are standardized betas of paths within the model)

	Intervention					
	1		2		3	
Path^a^	INT^b^	REC^c^	INT	REC	INT	REC
EFI → e-loyalty	ns^d^	ns	ns	ns	–.16	ns
EFE → e-loyalty	.28	.25	ns	.22	.42	.43
ENJ → e-loyalty	.26	.29	.19	.21	.20	.25
ACT → e-loyalty	.39	.33	.58	.40	.36	.26
EFE → ACT	.81		.71		.66	
TRU → ACT	ns		.16		.21	
*R^2^*	.73	.68	.56	.63	.65	.70
CFI^e^	.95		.95		.95	
TLI^f^	.94		.94		.94	
RMSEA^g^	.10		.09		.09	
SRMR^h^	.05		.06		.06	

^a^ EFI = efficiency; EFE = effectiveness; TRU = trustworthiness; ENJ = enjoyment; ACT = active trust.

^b^ Intention to visit again.

^c^ Recommend to others.

^d^ Not significant; all other paths are significant at the *P* = .05 level.

^e^ Comparative fit index.

^f^ Tucker-Lewis index.

^g^ Root mean square error of approximation.

^h^ Standardized root mean square residual.

**Table 5:** Results of extended model (figures are standardized betas of paths within the model)

	Intervention					
	1		2		3	
Path^a^	INT^b^	REC^c^	INT	REC	INT	REC
EFI → e-loyalty	ns^d^	ns	ns	ns	–.15	ns
EFE → e-loyalty	.27	.24	ns	.21	.40	.41
ENJ → e-loyalty	.23	.27	.14	.18	.17	.24
ACT → e-loyalty	.41	.35	.60	.42	.39	.27
EFE → ACT	.57		.51		.40	
TRU → ACT	ns		ns		ns	
ENJ → ACT	.37		.34		.47	
*R^2^*	.73	.68	.57	.64	.65	.70
CFI^e^	.95		.96		.96	
TLI^f^	.94		.95		.95	
RMSEA^g^	.09		.09		.09	
SRMR^h^	.05		.05		.05	

^a^ EFI = efficiency; EFE = effectiveness; TRU = trustworthiness; ENJ = enjoyment; ACT = active trust.

^b^ Intention to visit again.

^c^ Recommend to others.

^d^ Not significant; all other paths are significant at the *P* = .05 level.

^e^ Comparative fit index.

^f^ Tucker-Lewis index.

^g^ Root mean square error of approximation.

^h^ Standardized root mean square residual.

## Discussion

Our findings consistently demonstrate that effectiveness and enjoyment both had a positive effect on e-loyalty, which was mediated by active trust. The findings regarding effectiveness were anticipated and in line with previous research [28]. Mediation of the positive effect of enjoyment by active trust, however, was unanticipated. An explanation can be based on previous research demonstrating that enjoyment is related to cognitive perceptions [42]. Thus, cognitive perceptions might be a working mechanism for the positive effect of enjoyment on e-loyalty. Future research is needed to shed more light on the plausibility of this explanation, since this relationship can also be reversed: affective perceptions as a working mechanism leading to e-loyalty [43].

 Rejection of the hypothesis regarding the positive effect of efficiency on e-loyalty can be explained by the procedure used in this study. Efficiency refers to easy search of and access to the information provided. Participants, however, were not necessarily looking for information regarding the topic of the intervention websites to which they were exposed. Although participants could fill out the items regarding efficiency based on *whether* the intervention website at hand would be easy to search and access *if* they were looking for information at that intervention website, the lack of a need for information might explain the absence of evidence for a positive effect of efficiency. This could be solved by giving participants an assignment for which they have to study the intervention website thoroughly. The reason why we did not do this in the current study was to mimic a real-life situation in which people might review an intervention website when time limitations prevail [4]. This was reflected in this study as well, given the average time on website (range 1:28–3:10 minutes).

The lack of a relationship between trustworthiness and active trust in the structural equation models is puzzling. It might be that active trust by itself captures all the variance in e-loyalty that could be explained by trustworthiness. Since previous research demonstrated that active trust is the primary intermediate associated with e-loyalty [26,29], it might be that active trust reduces the possible impact of trustworthiness. This is contrary to previous research [28], however, and still does not explain the absence of a relationship between trustworthiness and active trust.

A final finding that deserves attention is that user perceptions and e-loyalty had low correlation with time on website. This can be explained by a confirmation bias [44]: since participants were told that they had to assess several websites, they might have been looking for evidence in line with their first impression, regardless of whether their impression was negative or positive. So, in the current setting the time spent on an intervention website is independent of user perceptions. Time on website may be related to user perceptions and e-loyalty if people explore an intervention website without any instructions.

In sum, although not all hypotheses were confirmed, this study clearly demonstrates that user perceptions (ie, effectiveness, enjoyment, and active trust) regarding e-loyalty are not important just in fields such as e-commerce, but also in the context of eHealth. The next question is how to improve user perceptions of intervention websites. To answer this question, characteristics of intervention websites need to be systematically manipulated, and these manipulations should be linked to user perceptions, and subsequently to e-loyalty. A possible variable to be manipulated is user control, defined as the voluntary and instrumental actions of a website visitor that influence the user experience [45,46]. The ability to control information flow increases one’s ability to explore and understand the structure of a website [47]. Nevertheless, one of the most common issues faced by visitors of websites is lack of user control [48]. This is awkward, given the wealth of literature (eg, McMillan and Hwang provide an overview [49]) documenting the importance of user control in shaping user experience [45,50]. Furthermore, previous research identified the role of user control (ie, freedom of choice) in attitude change [51] and intention to use [12,14]. The effect of user control on e-loyalty is in line with previous studies and is expected to be mediated through user perceptions [12,52]. Another characteristic to be manipulated in future research might be the use of tailoring strategies (eg, personalization, feedback) that have been shown to have a positive effect on intervention outcomes (in terms of health behaviors), which is related to intervention use [53].
